# Reproducibility of Non-Invasive Assessment of Skin Endothelial Function Using Laser Doppler Flowmetry and Laser Speckle Contrast Imaging

**DOI:** 10.1371/journal.pone.0061320

**Published:** 2013-04-19

**Authors:** Cyril Puissant, Pierre Abraham, Sylvain Durand, Anne Humeau-Heurtier, Sébastien Faure, Georges Lefthériotis, Pascal Rousseau, Guillaume Mahé

**Affiliations:** 1 Laboratory of Vascular Investigations, University Hospital, Angers, France; 2 Biologie Neurovasculaire et Mitochondriale Intégrée (BNMI) - Unité mixte UMR CNRS 6214/INSERM U 1083, LUNAM University, Medicine Faculty, Angers, France; 3 EA 4334 Motricity, Interactions, and Performance, LUNAM University, University du Maine, Le Mans, France; 4 LISA – Laboratoire d'Ingénierie des Systèmes Automatisés, LUNAM University, University of Angers, Angers, France; 5 INSERM U1063, Stress oxydant et pathologies métaboliques (SOPAM), LUNAM University, University of Angers, Angers, France; 6 Department of Plastic Surgery, University Hospital, Angers, France; University Medical Center Utrecht, The Netherlands

## Abstract

**Background:**

Endothelial dysfunction precedes atherosclerosis. Vasodilation induced by acetylcholine (ACh) is a specific test of endothelial function. Reproducibility of laser techniques such as laser-Doppler-flowmetry (LDF) and Laser-speckle-contrast-imaging (LSCI) to detect ACh vasodilation is debated and results expressions lack standardization. We aimed to study at a 7-day interval (i) the inter-subject reproducibility, (ii) the intra-subjects reproducibility, and (iii) the effect of the results expressions over variability.

**Methods and Results:**

Using LDF and LSCI simultaneously, we performed two different ACh-iontophoresis protocols. The maximal ACh vasodilation (peak-ACh) was expressed as absolute or normalized flow or conductance values. Inter-subject reproducibility was expressed as coefficient of variation (inter-CV,%). Intra-subject reproducibility was expressed as within subject coefficients of variation (intra-CV,%), and intra-class correlation coefficients (ICC). Fifteen healthy subjects were included. The inter-subject reproducibility of peak-ACh depended upon the expression of the results and ranged from 55% to 162% for LDF and from 17% to 83% for LSCI. The intra-subject reproducibility (intra-CV/ICC) of peak-ACh was reduced when assessed with LSCI compared to LDF no matter how the results were expressed and whatever the protocol used. The highest intra-subject reproducibility was found using LSCI. It was 18.7%/0.87 for a single current stimulation (expressed as cutaneous vascular conductance) and 11.4%/0.61 for multiple current stimulations (expressed as absolute value).

**Conclusion:**

ACh-iontophoresis coupled with LSCI is a promising test to assess endothelial function because it is reproducible, safe, and non-invasive. N°: NCT01664572.

## Introduction

Atherosclerosis is a major health problem in Western countries [1], [2]. It is commonly suggested that atherosclerosis process begins in childhood, progresses silently through a long preclinical stage depending on risk factors and genetic predisposition, and eventually manifests clinically, usually from middle age [3], [4]. Endothelial dysfunction precedes clinically detectable atherosclerosis and can also contribute to lesion development and later to clinical complications [2]. For several decades, endothelial dysfunction is considered as a marker of cardiovascular risk and could help to classify patients at high vascular risk [5]–[7]. As a result, assessment of endothelial function and dysfunction is of tremendous interest.

In the clinical perspective, the development of an easy, non-invasive test to routinely assess endothelial function is still required. Different invasive and non-invasive techniques have been developed to study endothelial function and have been widely reviewed elsewhere [5], [8], [9] but most of them are either technically demanding or not strictly a specific test of endothelial function [5].

An optimal tool for routine-use should, among others, be non-invasive, specific, and able to detect diseased patients [5], [10]. Several studies have demonstrated that endothelial function can be non-invasively, specifically assessed with acetylcholine (ACh) iontophoresis and is impaired in patients with vascular risk [11]–[16]. Further, the “optimal” tool must be reproducible. The reproducibility of single point laser Doppler flowmetry (LDF) is subject to debate but the recently available laser speckle contrast imaging (LSCI) has shown good reproducibility during local thermal hyperemia (LTH) and post-occlusive reactive hyperemia (PORH) (for specific details about LTH and PORH, please refer to [17]–[19]) [19]–[24]. Last, the protocols and results for an “optimal” tool should be standardized. Unfortunately, measurement expressions with laser techniques lack standardization [19], [25], [26]. Finally the “optimal” endothelial testing should be easy to perform. The measurement should be rapid to perform, acceptable for all subjects, old patients as well as young patients, and not painful.

Therefore, the goal of this study was to study in healthy subjects at a 7-day interval (i) the inter-subject reproducibility of ACh iontophoresis test, (ii) the intra-subjects reproducibility of ACh iontophoresis, and (iii) the effect of how the results of ACh iontophoresis test are expressed when comparing reproducibility during single and multiple stimulations using LDF and LSCI.

## Materials and Methods

### Study Population

Fifteen male or female volunteers (aged 18 years or older) without known cardiovascular disease were recruited in this study. Each subject gave his/her written informed consent prior to participation. This study received local institutional review board approval from the comité de protection des personnes (CPP) Ouest II, conformed to the Declaration of Helsinki, and was registered to the American National Institutes of Health database under reference N°: NCT01664572. The first subject was included July 2, 2012.

### Microvascular Recordings

All microvascular tests were performed with subjects resting supine in a temperature-controlled room (23±1°C). Tests were performed at least 2 hours after a meal. All tests were performed in the morning and at a same hour for each subject. All subjects had an acclimatization period longer than 15 min before the beginning of the cutaneous blood flow (CBF) acquisitions.

#### LDF recordings

The LDF relies on the Doppler effect. When photons of a laser light encounter moving particles (mainly red blood cells) in the studied tissues, the Doppler effect appears (modification of photon frequency). For LDF recordings, we used a laser Doppler flowmeter (Periflux PF 5000, Perimed, Järfälla, Sweden). Data of the CBF acquired by LDF were expressed as arbitrary units (a.u.) and recorded on a computer via an analogue to digital converter (Biopac System, Inc., California, USA) with a sample rate of 18 Hz, on 16-bits.

#### Laser speckle contrast imaging recordings

Recently, laser speckle contrast imaging (LSCI) has been commercialized [24], [27]. LSCI exploits the random speckle pattern generated by the illumination of the tissues under study by a coherent laser light. Motions of the particles in the tissues lead to changes in the speckle pattern that is imaged by a charge-coupled device (CCD). Due to the averaging of the speckle pattern over the exposure time (which is in the order of the speckle decorrelation-time, ms range), changes in speckle pattern give a blurred image (decorrelated pattern) and therefore an image with a reduced contrast area (usually defined as the ratio of the standard deviation of the intensity to the mean intensity of the speckle pattern). This blurring depends on the speed and volume of moving particles in the tissues: low contrast images come from a high amount of blood flow, and inversely, high contrast images come from a low amount of blood flow [28], [29]. LSCI recordings of the forearm CBF were performed using a 70-mW system (PeriCam PSI System ®, Perimed, Järfälla, Sweden) having a laser wavelength of 785 nm [30]. The sampling frequency was 18 Hz. The distance between the laser head and skin surface was fixed at 15 cm, according to our previous publication [31]. Images were stored on a computer and analyzed off-line. The signal amplitudes backscattered from the skin were calculated using the manufacturer’s software (PimSoft5.1 ®; Perimed, Järfälla, Sweden) before being exported to an Excel spreadsheet (Excel 2002 V3 ®, Microsoft USA). The software expresses recorded values in laser speckle perfusion units (LSPU).

### Iontophoresis

**Figure 1:**
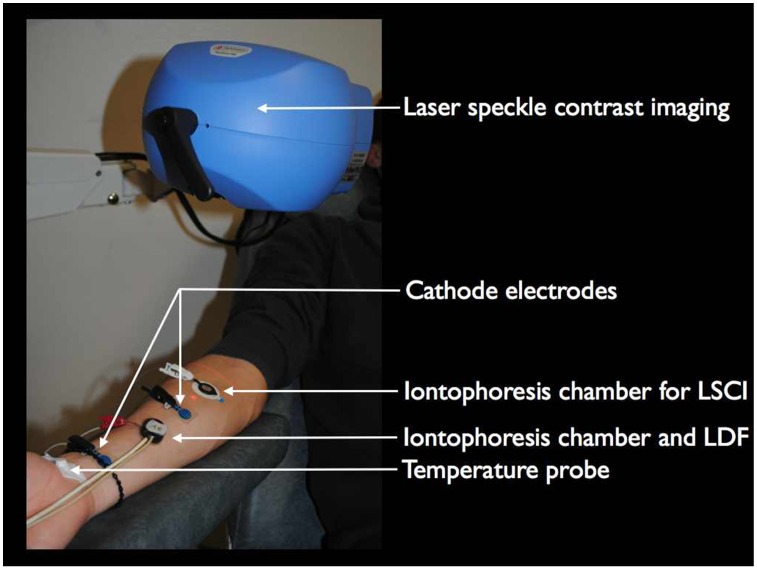
Typical recording using laser Doppler flowmetry (LDF) and laser speckle contrast imaging (LSCI) during protocol 1 and protocol 2.

Iontophoresis is a non-invasive method that drives a pharmacologically charged drug by electrorepulsion through the interstitium surrounding the blood vessels [32]. Transdermal iontophoresis of ACh (Sigma-Aldrich Corporation, L’Isle d’Abeau, France) was performed in the right forearm skin of each subject. Two iontophoreses were simultaneously performed: one with LDF and one with LSCI (Figure 1). Iontophoresis chambers (probe 481, Perimed, Jarfalla, Sweden, for LDF; and LI 611, Perimed Jarfalla, Sweden for LSCI) were randomly placed on subject skin and corresponded to anodes. Two cathode electrodes closed the electric circuit (Kendall, Mansfield, USA) and were fixed at 5 cm from the drug delivery chamber. Each iontophoresis chamber was filled with ACh (2%) dissolved in deionized water. The PeriIont Micropharmacology System (Perimed, Jarfalla, Sweden) delivered the current.

### Cardiovascular and Temperature Recordings

Blood pressure was recorded from the left middle finger (Nexfin, Bmeye, Amsterdam, Netherlands) with mean arterial pressure (MAP) obtained from the continuous blood pressure signal. The MAP signal was recorded at a sampling rate of 18 Hz using an analog to digital converter (MP150, Biopac sys, Goleta, USA) and analyzed offline using the Acknowledge ® software V3.5.4 (Biopac sys, Goleta, USA).

Local cutaneous temperature was measured on the right forearm using a surface thermocouple probe connected to an electronic thermometer (BAT-12®, Physitemp Instruments Inc., Clifton, NJ, USA).

### Protocols

All the subjects participated in 2 protocols.

#### Protocol 1: Inter-day reproducibility of endothelial function test with a single current stimulation

In protocol 1, the endothelial test consisted of an iontophoresis of ACh with a single current application (0.1 mA and 30 s) as previously proposed [26]. After a 2-minute resting period, a single current stimulation of 30 s was performed. Then, the signals were recorded for 20.5-min to record the late phase of ACh response. This procedure was recorded twice with a one-week interval: Day 1 (D1) and Day 7 (D7).

#### Protocol 2: Inter-day reproducibility of endothelial function test with multiple current stimulations

This second protocol was performed on a different day than the first protocol. In protocol 2, the endothelial test consisted of an iontophoresis of ACh with multiple current applications (four current stimulations of 0.1 mA and 10 s separated by a 2-min interval) as previously proposed [25]. After a rest of 2 min, four current stimulations of 10 s were performed. Then, a 20.5 min recording period was performed to record the late phase of ACh response (Day 1). This procedure was recorded twice with a one-week interval: D1 and D7.

### Data Analysis

The region of interest (ROI) is defined as the skin area of interest. The size of ROI was 20 mm^2^. Time of interest (TOI) is defined as the duration in seconds over which data (pixel values) are averaged [21]. It was set to 5 s for the maximal amplitude of ACh induced vasodilatation for LDF and LSCI. Baseline_(absolute)_ corresponds to the CBF measured before the first current stimulation and is expressed in a.u. when LDF was used and in laser speckle perfusion units (LSPU) when LSCI was used. Baseline_(CVC)_ corresponds to the CBF measured before the first current stimulation and expressed in Cutaneous Vascular Conductance (CVC). CVC is calculated as the laser signal value divided by the mean arterial pressure when the measurement is performed. CVC is expressed either in au/mmHg (for LDF) or in LSPU/mmHg (for LSCI). Baseline was measured with a ROI of 20 mm^2^ and a TOI of 10 s was chosen, when LSCI was used. For LDF, TOI was also of 10 s but no ROI can be defined with LDF. The following parameters of the maximal ACh vasodilation measurement obtained after current application are presented in Table 1.

**Table 1:** Different parameters of maximal vasodilation induced by acetylcholine iontophoresis (Peak ACh) studied in protocol 1 and protocol 2.

Parameters	Definitions	Units
Peak ACh_(absolute)_	the maximal value of vasodilation measured during the 2 min following the end of the current stimulation.	a.u. or LSPU
Time to peak	the time after the end of the current stimulation until the maximal ACh vasodilation is reached.	min
Peak ACh_(CVC)_	the maximal value of vasodilation measured during the 2 min following the end of the currentstimulation expressed in CVC.	au/mmHg or LSPU/mmHg
Peak ACh_(multiple CVC)_	the maximal value of vasodilation measured during the 2 min following the end of the current stimulationin multiple of baseline and is calculated as follows: Peak ACh_(multiple CVC)_ = Peak ACh_(CVC)_/Baseline_(CVC)_	No unit
Peak ACh_(increase CVC)_	the increase of vasodilation during the 2 min following the end of the current stimulation and iscalculated as follows: Peak ACh_(increase CVC)_ = [Peak ACh_(CVC)_−Baseline_(CVC)_]/Baseline_(CVC)_	No unit
Peak ACh_(increase Absolute)_	the maximum increase from baseline in absolute value during the 2 min following the end of the currentstimulation and is calculated as follows: Peak ACh_(increase absolute)_ = Peak ACh_(absolute)_−Baseline_(absolute)_	a.u. or LSPU

Cutaneous vascular conductance (CVC) is calculated as the laser signal value divided by the mean arterial pressure when the measurement is performed. When multiple current applications were performed, the number after each above parameters ranges from 1 to 4 corresponds to the number of current applications. For e.g., Peak 2 ACh_(CVC)_ corresponds to the maximal vasodilation obtained after the second current application and is expressed in CVC. min means minutes.

### Statistical Analysis

Results are expressed as mean±standard deviation (SD).

#### Concordance of the vasodilation patterns between D1 and D7

For each subject, the concordance of the vasodilation patterns between D1 and D7 was studied with cross-correlation analysis. For the cross-correlation analysis, the coefficient of correlation is a “r” value that, when reaching a maximum, is equal to the Pearson’s correlation coefficient. If the maximal “r” value is close to 1, the two signals are almost identical, whereas “r” is zero for two independent signals. The purpose of cross-correlation is to define whether signals have the same profile regardless of absolute values. Then for each subject we obtained a R_individual_. The mean of cross-correlation coefficients (R_individual_) between D1 and D7 was calculated as the mean of individual cross-correlation coefficients and compared with the Wilcoxon test. Wilcoxon test was used because R_individual_ distribution was not normally distributed when tested with Shapiro-Wilk test. Furthermore, the concordance of the average patterns (mean of individual vasodilation patterns) obtained with ACh iontophoresis between D1 and D7 was assessed using cross-correlation analysis (R_average_) for single current stimulation and multiple current stimulations.

#### Inter-subject reproducibility

The inter-subject reproducibility at a 7-day interval was evaluated by the coefficient of variations (inter-CV) calculated as the SD of CBF values of all subjects divide by the mean CBF of all subjects multiplied by 100. Comparisons of mean values of maximal vasodilation and time to peak between D1 and D7 obtained with LDF and LSCI were performed with the Wilcoxon test. A *p* value <0.05 was considered as statistically significant.

#### Intra-subject reproducibility

The intra-subject reproducibility at a 7-day interval was evaluated by the typical error of the estimate (TEE) of the peak of ACh as well as the coefficient of variations (intra-CV) according to the procedure proposed by Hopkins [33]. TEE was calculated as standard deviation of the paired differences/√2). For the calculation of intra-CV, data measured for each subject at D1 and D7 were log-transformed as proposed by Hopkins (http://www.sportsci.org/resource/stats/) [33]. Then intra-CV was calculated as 100*[e ^[(Standard deviation of the log-transformed paired differences/√2/100)]^−1]. The lower the intra-CV, the better the reproducibility. Intra-subject reproducibility of measurements was also assessed by intra-class correlation coefficients (ICC) [34]. ICC corresponds to a measure of agreement between test and re-test values of each measurement technique for D1 and D7 (Please refer to http://sportsci.org/ressource/stats/xrely.xls ) [33]. The ICC value ranges from 0.00 to 1.00, with values closer to 1.00 representing stronger reproducibility. ICC values >0.75, 0.75 to 0.60, 0.59 to 0.40, and <0.40 represent excellent, good, fair and poor agreements respectively [35]. Intra-CV and ICC are currently used to assess the reproducibility and represent additional information about the reproducibility of measurements [33]. Statistical analyses were performed with SPSS v17 (SPSS, Chicago, Illinois, USA).

#### Calculation of number of subjects

Number of subjects was calculated from ICCs by estimating the width of the 95% confidence interval for an expected correlation of 0.70 (between the lowest ICC value considered as acceptable, i.e. 0.40, and the highest, i.e. 1.00). Considering a two-sided interval, two measurements and a distance from correlation to limit *ω* of 0.3 (to remain between the range of fair to good agreements), the estimated sample size was 13. Considering the risk of missing data, we included 15 subjects in this study.

## Results

General characteristics of the population are presented in Table 2. Two out of six women (33%) took oral contraceptive treatment. For each protocol, no statistical difference was found between the experimental conditions at D1 and D7 (Table 2).

**Table 2:** General characteristics of the population and experimental conditions during protocol 1 and protocol 2.

	Population (n = 15)		
Age (years)	32±5		
Men (%)	9 (60%)		
BMI (kg/m2)	22.41±3.76		
Smoker (%)	1 (7%)		
During protocol 1 (single current stimulation)			
	D1	D7	*P*
Systolic blood pressure (mmHg)	111±10	117±11	NS
Diastolic blood pressure (mmHg)	70±7	72±6	NS
Heart rate (bpm)	62±10	62±11	NS
Skin temperature (°C)	32.9±1.4	33.3±1.1	NS
Laser head distance to skin (cm)	15.4±0.5	15.2±0.4	NS
During protocol 2 (multiple current stimulations)			
	D1	D7	*P*
Systolic blood pressure (mmHg)	119±9	116±8	NS
Diastolic blood pressure (mmHg)	68±7	72±5	NS
Heart rate (bpm)	62±10	62±9	NS
Skin temperature (°C)	32.7±1.6	32.0±2.0	NS
Laser head distance to skin (cm)	15.4±0.3	15.4±0.6	NS

BMI means Body mass index; bpm means beat per minute. Protocol 1 corresponds to ACh iontophoresis with a single current stimulation (0.1 mA, 30 s). Protocol 2 corresponds to ACh iontophoresis with a multiple current stimulations (four stimulations of 0.1 mA, 10 s with a 2 minute free interval). Protocol 1 and 2 were performed on separated days. Laser head distance was the distance between the laser speckle head and the skin. P means p value. NS means non significant.

### Concordance of the Vasodilation Patterns between D1 and D7

**Figure 2:**
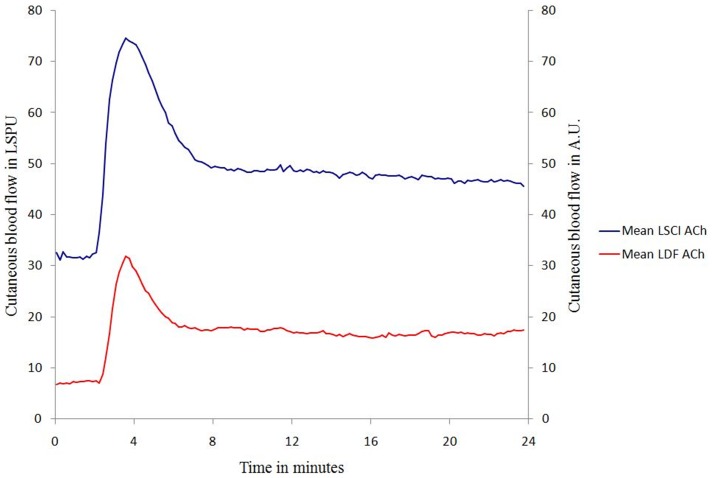
Mean vasodilation patterns obtained with ACh iontophoresis between D1 and D7 for each technique (LDF: laser Doppler flowmetry and LSCI: laser speckle contrast imaging) using a single current stimulation (0.1 mA and 30 s).

**Figure 3:**
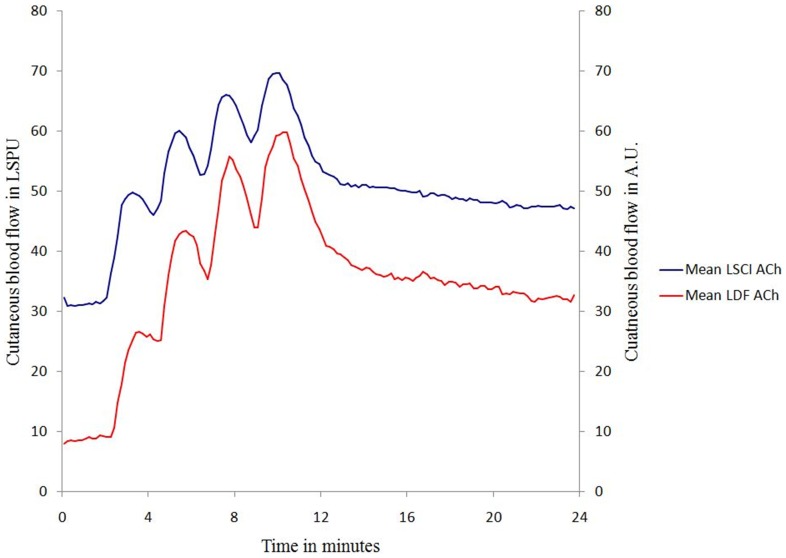
Mean vasodilation patterns obtained with ACh iontophoresis between D1 and D7 for each technique (LDF: laser Doppler flowmetry and LSCI: laser speckle contrast imaging) using multiple current stimulations (0.1 mA and 10 s separated with a 2-minute interval).

The typical vasodilation patterns obtained by ACh iontophoresis either with a single current stimulation (protocol 1) or with multiple current stimulations (protocol 2) are presented in figure 2 & 3 for LDF and LSCI.

#### Protocol 1 (single current stimulation)

The R_individual_ between D1 and D7 were 0.68 (0.24), and 0.74 (0.25) for LDF and LSCI, respectively. No statistical difference was found between R_individual_ for LDF compared with R_individual_ for LSCI. The R_average_ between D1 and D7 were 0.82 using LDF whereas it was 0.98 using LSCI.

#### Protocol 2 (multiple current stimulation)

The R_individual_ between D1 and D7 were 0.65 (0.16) and 0.76 (0.20) for LDF and LSCI, respectively. This difference was statistically significant (p = 0.036). The R_average_ between D1 and D7 was 0.94 using LDF whereas it was 0.98 using LSCI.

### Inter-subject Reproducibility

Mean values of the maximal ACh vasodilation measured at D1 compared to mean values of the maximal ACh vasodilation measured at D7 are presented in Table 3 for LDF and LSCI. No statistical difference was found between mean values of ACh measured at D1 and mean values of ACh measured at D7, which every way the results were expressed for both techniques and protocols. However, inter-subject reproducibility (inter-CVs) was higher using LDF than using LSCI no matter how peaks ACh were expressed.

**Table 3:** Mean values of ACh peak measured in the whole population either with LDF or LSCI at day 1 (D1) and day 7 (D7) in protocols 1 and 2.

	Single current stimulation (Protocol 1)							
	LDF				LSCI			
	D1	Inter-CV	D7	Inter-CV	D1	Inter-CV	D7	Inter-CV
Baseline_(CVC)_	0.09±0.04	44%	0.09±0.07	78%	0.37±0.09	24%	0.37±0.10	27%
Time to Peak (min)	1.20±0.47	39%	1.22±0.44	36%	0.93±0.63	68%	1.02±0.55	54%
Peak ACh_(absolute)_	34.0±20.4	60%	41.1±35.1	85%	73.9±25.7	35%	72.5±23.7	33%
Peak ACh_(CVC)_	0.38±0.21	55%	0.42±0.36	86%	0.84±0.33	39%	0.80±0.28	35%
Peak ACh_(multiple)_	5.5±5.9	107%	5.7±4.0	70%	2.3±0.7	30%	2.2±0.6	27%
Peak ACh_(increase CVC)_	4.5±5.9	131%	4.7±4.0	85%	1.3±0.7	54%	1.2±0.6	50%
Peak ACh_(increase absolute)_	26.6±19.0	71%	32.0±31.3	98%	42.6±22.5	53%	40.3±21.1	52%
	**Multiple current stimulations (Protocol 2)**							
	**LDF**				**LSCI**			
	**D1**	**Inter-CV**	**D7**	**Inter-CV**	**D1**	**Inter-CV**	**D7**	**Inter-CV**
Baseline_(CVC)_	0.10±0.05	50%	0.11±0.07	64%	0.38±0.07	18%	0.37±0.10	27%
Time to Peak 1 (min)	1.12±0.54	48%	1.41±0.41	29%	0.59±0.37	63%	0.82±0.25	30%
Peak 1 ACh_(absolute)_	25.8±29.4	114%	30.1±34.2	114%	55.5±13.7	25%	50.5±10.3	20%
Peak 1 ACh_(CVC)_	0.28±0.33	118%	0.35±0.40	114%	0.62±0.18	29%	0.58±0.16	28%
Peak 1 ACh_(multiple)_	3.1±3.0	97%	2.9±2.0	69%	1.6±0.5	31%	1.6±0.4	25%
Peak 1 ACh_(increase CVC)_	2.1±3.0	143%	1.9±2.0	105%	0.6±0.5	83%	0.6±0.4	66%
Peak 1 ACh_(increase absolute)_	17.6±28.5	162%	20.9±30.5	146%	21.8±13.4	61%	20.1±9.4	47%
Time to Peak 2 (min)	3.51±0.54	15%	3.52±0.39	11%	2.96±0.24	8%	2.98±0.28	9%
Peak 2 ACh_(absolute)_	36.4±38.1	105%	50.5±46.4	92%	63.8±12.5	20%	59.6±11.7	19%
Peak 2 ACh_(CVC)_	0.39±0.44	113%	0.58±0.56	97%	0.71±0.18	25%	0.69±0.19	28%
Peak 2 ACh_(multiple)_	4.5±4.1	91%	5.7±5.6	98%	1.9±0.5	26%	1.9±0.6	32%
Peak 2 ACh_(increase CVC)_	3.5±4.1	117%	4.7±5.6	119%	0.9±0.5	56%	0.9±0.6	67%
Peak 2 ACh_(increase absolute)_	28.2±37.1	132%	41.2±42.9	104%	31.0±12.7	41%	29.2±12.2	42%
Time to Peak 3 (min)	5.79±0.56	10%	5.70±0.31	5%	5.17±0.17	3%	5.29±0.27	5%
Peak 3 ACh_(absolute)_	43.1±43.2	100%	61.1±58.7	96%	69.3±11.9	17%	65.3±11.0	17%
Peak 3 ACh_(CVC)_	0.45±0.45	100%	0.71±0.71	100%	0.76±0.17	22%	0.74±0.16	22%
Peak 3 ACh_(multiple)_	5.2±4.4	85%	6.9±8.1	117%	2.0±0.5	25%	2.1±0.6	29%
Peak 3 ACh_(increase CVC)_	4.2±4.4	105%	5.9±8.1	137%	1.0±0.5	50%	1.1±0.6	55%
Peak 3 ACh_(increase absolute)_	34.9±42.4	121%	51.9±55.1	106%	36.5±12.4	34%	34.9±12.6	36%
Time to Peak 4 (min)	8.11±0.81	10%	8.08±0.58	7%	7.25±0.45	6%	7.53±0.28	4%
Peak 4 ACh_(absolute)_	48.3±43.1	89%	69.6±60.0	86%	72.4±13.4	19%	69.7±11.7	17%
Peak 4 ACh_(CVC)_	0.50±0.45	90%	0.82±0.71	87%	0.80±0.20	25%	0.81±0.21	26%
Peak 4 ACh_(multiple)_	5.9±4.5	76%	8.4±9.7	115%	2.1±0.5	24%	2.3±0.7	30%
Peak 4 ACh_(increase CVC)_	4.9±4.5	92%	7.4±9.7	131%	1.1±0.5	45%	1.3±0.7	54%
Peak 4 ACh_(increase absolute)_	40.1±43.3	108%	60.4±56.7	94%	39.6±14.0	35%	39.3±13.4	34%

The inter-subject variability at a 7-day interval was evaluated by the coefficient of variations (inter-CV) calculated as the SD of CBF values of all subjects divide by the mean CBF of all subjects multiplied by 100. The parameters of the maximal ACh vasodilation measurement obtained after current application are presented in Table 1. Which every way the results were expressed for both techniques, no statistical difference was found between mean values of ACh measured at D1 and mean values of ACh measured at D7. Time to peak corresponds to the time after the end of the current stimulation until the maximal acetylcholine vasodilation is reached.

### Intra-subject Reproducibility

Intra-subject reproducibility of maximal ACh vasodilation at a 7-day interval using LDF and LSCI are presented in table 4 for protocol 1 and table 5 for protocol 2. For both techniques and both protocols, CVs and ICCs were depending on the way of expressing the results. For LDF, CVs ranged from 50.1% to 323.2% and ICCs ranged from 0.01 to 0.81. For LSCI, CVs ranged from 11.4% to 194.2% and ICCs ranged from 0.04 to 0.87.

**Table 4:** Intra-subject reproducibility at a 7-day interval of ACh vasodilation with a single current stimulation measured either using LSCI or LDF.

	Single current stimulation					
	LDF			LSCI		
	TEE	Intra-CV (%)	ICC	TEE	Intra-CV (%)	ICC
Baseline_(absolute)_	5.00 [3.66–7.89]	93.9 [62.4–184.1]	0.03 [−0.48–0.52]	4.18 [3.06–6.59]	14.9 [10.7–24.6]	0.65 [0.22–0.87]
Peak ACh_(absolute)_	28.74 [21.04–45.32]	123.2 [80.0–254.6]	−0,01 [−0.50–0.49]	12.55 [9.19–19.80]	23.4 [16.6–39.3]	0.77 [0.44–0.92]
Peak ACh_(CVC)_	0.27 [0.20–0.43]	116.2 [74.9–246.2]	0.16 [−0.37–0.61]	0.12 [0.09–0.19]	18.7 [13.4–31.0]	0.87 [0.65–0.95]
Peak ACh_(multiple CVC)_	3.87 [2.83–6.11]	80.0 [53.8–152.8]	0.43 [−0.09–0.76]	0.43 [0.32–0.68]	28.2 [19.9–48.0]	0.64 [0.20–0.86]
Peak ACh_(increase CVC)_	3.87 [2.83–6.11]	139.2 [89.4–295.7]	0.43 [−0.09–0.76]	0.36 [0.26–0.56]	194.2 [120.4–448.5]	0.74 [0.39–0.91]
Peak ACh_(increase absolute)_	25.56[18.71–40.30]	188.8 [117.4–432.5]	0.03 [−0.48–0.52]	12.63 [9.25–19.93]	121.0 [78.7–249.1]	0.71 [0.34–0.89]

The parameters of the maximal ACh vasodilation measurement obtained after current application are presented in Table 1. Time to peak corresponds to the time after the end of the current stimulation until the maximal acetylcholine vasodilation is reached. Typical error of the estimate (TEE) of the cutaneous blood flow expressed in au or LSPU when (absolute) is mentioned and in au/mmHg or LSPU/mmHg when CVC is mentioned. Intra-CV means intra-subject coefficient of variation. ICC means intra-class correlation. Results are mean with 95% confidence interval [95%]. The lower the intra-CV, the better the reproducibility. ICC values of >0.75, 0.75 to 0.60, 0.59 to 0.40, and <0.40 represent excellent, good, fair and poor agreements respectively.

**Table 5:** Intra-subject reproducibility at a 7-day interval of ACh vasodilation with multiple current stimulations measured either using LSCI or LDF.

	Multiple current stimulations (Protocol 2)					
	LDF			LSCI		
	TEE	Intra-CV (%)	ICC	TEE	Intra-CV (%)	ICC
Baseline_(absolute)_	4.62 [3.38–7.29]	77.1 [52.0–146.3]	0.13 [−0.39–0.59]	2.93 [2.15–4.62]	9.9 [7.1–16.0]	0.69 [0.29–0.88]
Peak 1 ACh_(absolute)_	33.16 [24.28–52.29]	147.4 [94.1–317.2]	−0.09 [–0.56–0.43]	11.86 [8.68–18.71]	24.4 [17.3–41.1]	0.04 [−0.46–0.53]
Peak 1 ACh_(CVC)_	0.38 [0.28–0.60]	148.5 [94.7–320.3]	−0.09 [–0.56–0.42]	0.16 [0.12–0.26]	28.2 [20.0–48.0]	0.10 [–0.42–0.57]
Peak 1 ACh_(multiple CVC)_	1.92 [1.40–3.02]	70.7 [47.9–132.4]	0.48 [−0.02–0.79]	0.40 [0.29–0.63]	27.1 [19.2–46.0]	0.26 [–0.27–0.67]
Peak 1 ACh_(increase CVC)_	1.92 [1.40–3.02]	323.2 [187.6–873.7]	0.48 [−0.02–0.79]	0.40 [0.29–0.63]	162.8 [102.9–359.1]	0.26 [–0.27–0.67]
Peak 1 ACh_(increase absolute)_	29.66 [21.72–46.78]	315 [180.6–890.2]	−0.01 [–0.51–0.49]	10.33 [7.56–16.29]	64.7 [43.6–123.3]	0.22 [−0.31–0.65]
Peak 2 ACh_(absolute)_	35.84 [26.24–56.52]	114.6 [74.9–233.4]	0.31 [–0.22–0.70]	11.03 [8.07–17.39]	20.6 [14.7–34.3]	0.18 [–0.35–0.62]
Peak 2 ACh_(CVC)_	0.44 [0.32–0.70]	115.6 [75.5–236.0]	0.25 [−0.29–0.66]	0.14 [0.11–0.23]	24.9 [17.6–41.9]	0.41 [−0.11–0.75]
Peak 2 ACh_(multiple CVC)_	2.27 [1.66–3.59]	50.1 [34.6–89.7]	0.81 [0.53–0.93]	0.41 [0.30–0.64]	24.0 [17.1–40.4]	0.50 [0.01–0.80]
Peak 2 ACh_(increase CVC)_	2.27 [1.66–3.59]	211.1 [129.5–498.8]	0.81 [0.53–0.93]	0.41 [0.30–0.64]	85.4[57.2–164.9]	0.50 [0.01–0.80]
Peak 2 ACh_(increase absolute)_	32.19 [23.56–50.76]	299.4 [172.9–830.9]	0.38 [−0.14–0.74]	9.69 [7.10–15.29]	46.4 [31.8–84.8]	0.42 [−0.10–0.76]
Peak 3 ACh_(absolute)_	37.30 [27.31–58.83]	103.8 [68.4–207.4]	0.51 [0.01–0.80]	7.45 [5.46–11.75]	11.4 [8.2–18.5]	0.61 [0.16–0.85]
Peak 3 ACh_(CVC)_	0.48 [0.35–0.76]	109.3 [71.7–220.6]	0.38 [–0.15–0.74]	0.11 [0.08–0.17]	15.4 [11.1–25.3]	0.62 [0.17–0.85]
Peak 3 ACh_(multiple CVC)_	3.65 [2.67–5.76]	53.5 [36.8–96.5]	0.72 [0.34–0.89]	0.38 [0.28–0.60]	21.3 [15.2–35.6]	0.56 [0.08–0.83]
Peak 3 ACh_(increase CVC)_	3.65 [2.67–5.76]	120.8 [78.6–248.7]	0.72 [0.34–0.89]	0.38 [0.28–0.60]	98.2 [65.0–194.1]	0.56 [0.08–0.83]
Peak 3 ACh_(increase absolute)_	33.66 [24.64–53.08]	179.6 [110.7–424.2]	0.56 [0.09–0.83]	6.40 [4.69–10.10]	18.6 [13.2–31.7]	0.76 [0.43–0.91]
Peak 4 ACh_(absolute)_	35.52 [26.01–56.02]	107.9 [70.9–217.2]	0.58 [0.11–0.83]	8.69 [6.36–13.70]	12.9 [9.3–21.1]	0.56 [0.09–0.83]
Peak 4 ACh_(CVC)_	0.46 [0.34–0.73]	119.6 [77.9–245.7]	0.43 [−0.09–0.76]	0.16 [0.12–0.25]	21.5 [15.3–35.9]	0.42 [−0.10–0.76]
Peak 4 ACh_(multiple CVC)_	4.84 [3.54–7.63]	63.3 [43.2–116.7]	0.62 [0.18–0.85]	0.40 [0.29–0.63]	20.5 [14.6–34.2]	0.59 [0.13–0.84]
Peak 4 ACh_(increase CVC)_	4.84 [3.54–7.63]	136.2 [87.6–287.9]	0.62 [0.18–0.85]	0.40 [0.29–0.63]	54.1 [37.3–97.8]	0.59 [0.13–0.84]
Peak 4 ACh_(increase absolute)_	31.70 [23.21–50.00]	174.3 [107.8–408.2]	0.64 [0.20–0.86]	7.41 [5.43–11.69]	23.0 [16.2–39.6]	0.74 [0.38–0.90]

The parameters of the maximal ACh vasodilation measurement obtained after current application are presented in Table 1. Time to peak corresponds to the time after the end of the current stimulation until the maximal acetylcholine vasodilation is reached. Typical error of the estimate (TEE) of the cutaneous blood flow expressed in au or LSPU when (absolute) is mentioned and in au/mmHg or LSPU/mmHg when CVC is mentioned. Intra-CV means intra-subject coefficient of variation. ICC means intra-class correlation. Results are mean with 95% confidence interval [95%]. The lower the intra-CV, the better the reproducibility. ICC values of >0.75, 0.75 to 0.60, 0.59 to 0.40, and <0.40 represent excellent, good, fair and poor agreements respectively.

In protocol 1, using LDF, the best CV was 80.0% [53.8–152.8] and was obtained when results were expressed in peak ACh_(multiple_ _CVC)_ whereas the best CV was 18.7% [13.4–31.0] when results were expressed in peak ACh_(CVC)_ with LSCI.

In protocol 2, using the LDF, the best CV was 50.1% [34.6–89.7]; it was obtained when results were expressed in peak ACh_(multiple CVC),_ whereas using LSCI, the best CV was 11.4% [8.2–18.5]; it was obtained when results were expressed in peak ACh_(absolute)_.

Using LDF, intra-subject reproducibility was always lower than using LSCI when results were expressed either in peak ACh_(absolute)_ or in peak ACh_(CVC)_ or in peak ACh_(multiple CVC)_. Which every way the results were expressed, the CVs obtained with LDF were higher than those obtained using LSCI. Only the intra-subject reproducibility measured with LSCI fulfilled both conditions (i.e., low intra-CV and ICC>0.40) but this depended on the way of expressing the results.

## Discussion

This study provides original results about the inter-day reproducibility of laser techniques (LDF and LSCI) in order to assess endothelial function in clinical routine or clinical research. The major findings are: (i) the typical vasodilation patterns induced by ACh iontophoresis measured by LDF and LSCI are highly correlated at a 7-day interval; (ii) the inter-subject reproducibility is better when using LSCI; (iii) the intra-subject reproducibility is highly improved with LSCI and depends on the way of expressing the results.

First, we confirmed the typical vasodilation patterns previously described by Durand *et al.*, by Debbabi *et al*. and Sauvet *et al.* using LDF [25], [26], [36]. Both protocols are of interest and are known to induce no current-induced vasodilation when ACh iontophoresis is performed with deionized water as a vehicle [26], [36]. Indeed it has been suggested that some vehicles (NaCl, tap water…) as well as some protocols and charge densities (relying on the chamber surface and expressed in mC/cm^2^) might induce non-specific vasodilation, which can be a confounding factor when intophoresis is used [32], [37]–[39]. However, using our protocols, it has been shown that ACh iontophoresis with a single current application (protocol 1) induces an endothelial-dependent vasodilatory response, which is biphasic with a rapid peak relying on muscarinic receptor M_3_ and a late plateau, which involves muscarinic receptor and prostaglandins [26]. Protocols with multiple current stimulations have been developed to reduce the current effect by applying low intensity at set time interval and may mimic the technique of in-vitro vessel preparations [32]. Further using multiple current stimulations, a strong linear relationship (r = 0.92; p<0.0001) was found between ACh iontophoresis (third peak) and flow-mediated dilation [25]. Although it is well admitted that vasodilation induced by ACh iontophoresis is endothelial dependent, the specific intra-cellular pathways, which are involved in such vasodilation are discussed [19], [40]. Three pathways have been evoked: NO pathway, prostanoids pathway and endothelium derived hyperpolarizating factor (EDHF) pathway [19], [41], [42].

Second, LDF and LSCI showed a high concordance between D1 and D7 with a statistically lower concordance with LDF compared with LSCI in protocol 2. Therefore, kinetic of the vasodilation involved in the ACh iontophoresis is similar at a 7 day-interval. Moreover we demonstrated that peak ACh is rapidly obtained in less than 10 minutes for both protocols showing the interest of such endothelial testing in clinical routine. The duration of endothelial function testing is a key point because this determines the applicability of a test in clinical routine.As compared with the most used non-invasive endothelial function tests (peripheral arterial tonometry and Flow Mediated Dilation (FMD)), ACh iontophoresis measured either with LDF or LSCI appears shorter in term of duration measurement without taking into account the time for set-up. Endothelial function assessment with peripheral arterial tonometry is performed in approximately 15 minutes (http://www.itamar-medical.com/EndoPAT/FAQ.html) whereas with FMD the measurement lasts longer than 20 min [5], [43]. However as for laser techniques, peripheral arterial tonometry can be performed by nurses contrary to FMD, which necessitates highly trained technicians [5].

Third, this study confirmed that the inter-subject reproducibility is improved when LSCI is used as compared with LDF. When LSCI is used, inter-CVs are roughly twice lower than inter-CVs obtained using LDF. This is found no matter how the results are expressed. Therefore, when calculating the sample size for a study, fewer patients will be required when LSCI is used as compared with a study using LDF. For example, when performing a study which compares endothelial function expressed in Peak ACh_CVC_ of two independent groups, where the anticipated difference is 20%, the sample size required for an α = 0.05 and a power of 80% would be 121 per group using LDF and 62 per group using LSCI when single current stimulation is performed. In case of performing multiple current stimulations and using the third peak_CVC_, the number of subjects in each group would be 394 using LDF and 21 using LSCI. The important decrease of the sample size when LSCI is used is in accordance with the sample size calculation found when the laser Doppler perfusion imaging is used [44].

Fourth, we found that the intra-subject reproducibility was improved with LSCI compared with LDF. This corroborates other results, which compared other microvascular tests measured with LSCI and LDF. Roustit *et al*. have shown that local thermal hyperaemia peak had a CV of 15% (when data were expressed as CVC) and the post-occlusive reactive hyperaemia a CV of 8% (when data were expressed as CVC) using LSCI whereas CV was of 40% (when data were expressed as CVC) and 30% (when data were expressed as CVC) when LDF was used, respectively [20]. It has been suggested that LSCI significantly improves the reproducibility of the measurement as compared with the LDF technique, since the measurements are averaged over large surface areas, thereby reducing the spatial variability of cutaneous microcirculation [20], [21]. However, intra-subject reproducibility of ACh vasodilation was nearly similar with the reproducibility found using laser Doppler perfusion imaging, where the CV was approximately 20% between two different days in two different sites [45], [46]. For laser Doppler perfusion imaging, the CV was even lower than 10% for a same site between two different days [40], [46]. But, the authors marked the site of measurement with permanent ink [46]. We did not make this choice because marking the site of measurement with permanent ink is not possible when long follow-up of subjects or patients is performed. Further, it has been proposed that cleaning by using an adhesive tape to remove superficial dead layer of skin or washed with alcohol followed by sterile water improved the reproducibility. We did not perform this since we wanted to avoid any potential bias between the two sites of measurements [13], [45], [47]. Moreover, we did not used EMLA cream (lidocaïne+prilocaine) since no current induced vasodilation was found with both protocols (data not shown). For FMD and peripheral arterial tonometry, inter-day CVs were approximately 11% for both techniques [48], [49]. This is lower than our results found for LSCI but the CV and ICC are not the only points to take into account for defining a good test. Indeed, laser techniques fulfill other important points which are: the ease to use the technique, its cost, its safety, its duration, the fact that the technique is non-invasive, the fact that it has been suggested to reflect coronary lesions, and the fact that it could measure reversibility with interventions [5], [11], [16], [50], [51]. These two last points warrant further investigations.

Last, this study showed that the way of expressing the results has an important effect over the variability of the peak ACh. For both protocols (1 & 2), expressing the results in peak ACh_(increase CVC)_ and peak ACh_(increase absolute)_ result in a worse reproducibility as compared with the other ways of expressing the results. Further, as it has been suggested by several authors, expressing the results in CVC is better than expressing the results in absolute values in order to take into account differences and variations in blood pressure [40], [52]. Considering this, the best intra-subject reproducibility was found using LSCI and was for a single current stimulation (18.7%/0.87), and for multiple current stimulations (15.4%/0.62).

### Study Limitations

The main limitation of this study is that we did not show that the peak ACh is sensitive to health status changes or treatments. However, using laser flowmetry these points have been previously shown [16], [53], [54]. Another point is to assess the same issue in another population and specifically in diseased subjects. Further, the stage of menstrual cycle was not controlled in this study. This might have influenced the reproducibility since the role of female hormonal status over skin reactivity is discussed [55], [56]. Moreover, we cannot exclude that larger ROIs will have conducted to a superior reproducibility but larger ROIs do not facilitate an easy subtraction of movement artifacts because it is time-consuming to displace the ROI image by image for keeping the ROI inside the iontophoresis chamber zone. Last, extreme sensitivity of LSCI to movements might appear a limitation of the technique in routine, but recent reports suggest that a reference zero flow patch may be successfully used to get rid of movement artifacts in LSCI recordings [57], [58].

### Conclusion

In order to assess endothelial function in clinical routine and human research, the use of LSCI, appears preferable to the use of LDF in term of reproducibility. The simplicity of the use of LSCI, and its excellent reproducibility should facilitate future studies dealing with cardiovascular risk factor management and patient outcomes.
